# Elimination of *Aspergillus fumigatus* conidia from the airways of mice with allergic airway inflammation

**DOI:** 10.1186/1465-9921-14-78

**Published:** 2013-07-27

**Authors:** Marina A Shevchenko, Elena L Bolkhovitina, Ekaterina A Servuli, Alexander M Sapozhnikov

**Affiliations:** 1 Department of Immunology, Laboratory of Cell Interactions, Shemyakin-Ovchinnikov Institute of Bioorganic Chemistry, Russian Academy of Sciences, Miklukho-Maklaya St. 16/10, 117997 Moscow, Russia; 2 Faculty of Postgraduate Education, Pirogov Russian National Research Medical University, Ministry of Health, Ostrovitianov St. 1, 117997 Moscow, Russia

**Keywords:** Allergic airway inflammation, *Aspergillus fumigatus*, Neutrophils, Mucosal dendritic cells, Conducting airway, Confocal laser scanning microscopy

## Abstract

**Background:**

*Aspergillus fumigatus* conidia can exacerbate asthma symptoms. Phagocytosis of conidia is a principal component of the host antifungal defense. We investigated whether allergic airway inflammation (AAI) affects the ability of phagocytic cells in the airways to internalize the resting fungal spores.

**Methods:**

Using BALB/c mice with experimentally induced AAI, we tested the ability of neutrophils, macrophages, and dendritic cells to internalize *A. fumigatus* conidia at various anatomical locations. We used light microscopy and differential cell and conidium counts to determine the ingestion potential of neutrophils and macrophages present in bronchoalveolar lavage (BAL). To identify phagocyte-conidia interactions in conducting airways, conidia labeled with tetramethylrhodamine-(5-(and-6))-isothiocyanate were administered to the oropharyngeal cavity of mice. Confocal microscopy was used to quantify the ingestion potential of Ly-6G^+^ neutrophils and MHC II^+^ antigen-presenting cells located in the intraepithelial and subepithelial areas of conducting airways.

**Results:**

Allergen challenge induced transient neutrophil recruitment to the airways. Application of *A. fumigatus* conidia at the acute phase of AAI provoked recurrent neutrophil infiltration, and consequently increased the number and the ingestion potential of the airway neutrophils. In the absence of recurrent allergen or conidia provocation, both the ingestion potential and the number of BAL neutrophils decreased. As a result, conidia were primarily internalized by alveolar macrophages in both AAI and control mice at 24 hours post-inhalation. Transient influx of neutrophils to conducting airways shortly after conidial application was observed in mice with AAI. In addition, the ingestion potential of conducting airway neutrophils in mice with induced asthma exceeded that of control mice. Although the number of neutrophils subsequently decreased, the ingestion capacity remained elevated in AAI mice, even at 24 hours post-conidia application.

**Conclusions:**

Aspiration of allergen to sensitized mice enhanced the ingestion potential of conducting airway neutrophils. Such activation primes neutrophils so that they are sufficient to control dissemination of non-germinating *A. fumigatus* conidia. At the same time, it can be a reason for the development of sensitivity to fungi and subsequent asthma exacerbation.

## Background

*Aspergillus* are among the most common airborne fungi [1]. Fungal spores are present in both outdoor and indoor air, primarily in a resting or dormant state [2]. The inhalation of *Aspergillus* conidia can induce allergic sensitization and exacerbate the allergic airway inflammation (AAI) [3,4]. Moreover, cardinal features of allergic asthma, such as IL-13-mediated mucus production and goblet cell hyperplasia, are important for fungal clearance and antifungal host defense [5,6]. Despite the allergic potential of fungi, only 13% of asthmatic patients show sensitization to *Aspergillus* species [7]. The low sensitivity can be partially explained by the resting or dormant state of the inhaled conidia [2,8]. A hydrophobic surface layer covers resting conidia and masks the fungal molecular patterns, thereby protecting conidia from immune system recognition [8,9]. Such inertness allows a tolerance to airborne fungi by immunocompetent hosts [10].

Although resting *A. fumigatus* conidia do not activate the immune system [8,11], they can enhance pre-existing AAI. Thus, inactivated *A. fumigatus* spores trigger Th2 cytokine production by CD4^+^ T-cells that have been previously stimulated with antigen-pulsed antigen-presenting cells (APCs) [12]. Fukushima et al. [4] injected mice exhibiting mite-induced AAI with heat inactivated conidia, which mimic the resting fungal spores, and observed increased bronchoalveolar lavage (BAL) eosinophil number, as well as IL-5 and IL-13 expression by the lung cells. Hence, daily exposure to ubiquitous dormant *A. fumigatus* conidia induces tolerance in healthy people, but can provoke exacerbation in asthmatics.

Neutrophils are the predominant inflammatory cells that infiltrate airways during acute exacerbation of asthma [13]. Neutrophils also play an essential role in antifungal defense [14,15]. They rapidly migrate to the infected lungs and suppress conidial germination through phagocytosis or extracellular trap formation [16,17]. Allergen challenge also induces transient neutrophil recruitment to the airways [18,19]. However, neutrophils from the sputum of asthmatics produce lower levels of IL-8, IL-1β, and TNF-α, and show reduced levels of TLR4 mRNA expression [20]. Therefore, the alteration of neutrophil functions in asthma can be a trigger for tolerance-to-sensitivity shift.

To determine whether allergic asthma alters the ingestion activity of phagocytic airway cells, we examined the ability of neutrophils, macrophages, and dendritic cells (DCs) to control resting *A. fumigatus* conidial dissemination in the airways of mice with AAI. To accomplish this, mice with ovalbumin (OVA)-induced AAI were inoculated via inhalation with fixed *A. fumigatus* conidia at the acute stage of inflammation. Analysis of the phagocytic cells and conidial interactions at different time points, and in various anatomical compartments of the respiratory tract, enabled us to identify the elevated ingestion potential of conducting airway neutrophils as a sustained attribute of AAI, independent from the recruited neutrophil number.

## Methods

### Animals

Eight-week-old BALB/c mice (Charles River Laboratories, Sulzfeld, Germany, and the Central Laboratory of the Animal Breeding Facility, Russian Academy of Medical Sciences, Moscow, Russia) were used in the study. Animals were fed with OVA-free laboratory food and tap water *ad libitum*, and held in regular 12-hour dark:light cycles at 22°C. Animal experiments were performed in concordance with the German animal protection law, under a protocol approved by the appropriate governmental authority (Niedersächsisches Landesamt für Verbraucherschutz und Lebensmittelsicherheit), or in accordance with the Guide for the Care and Use of Laboratory Animals, under a protocol approved by the Institutional Animal Care and Use Committee of the Shemyakin-Ovchinnikov Institute of Bioorganic Chemistry RAS.

### *A. fumigatus* strain, media, and growth conditions

*A. fumigatus* clinical isolate D141 [21] was used in the present study. The fungus was grown at 37°C on *Aspergillus* minimal medium (AMM) containing 1% D-glucose as the carbon source and 70 mM NaNO_3_ as the nitrogen source. A fungal suspension was transferred to AMM agar plates and incubated for 3 days at 37°C. Harvested conidia were fixed overnight with 3% paraformaldehyde (Sigma-Aldrich, St. Louis, MO, USA), washed twice with PBS, and then labeled with tetramethylrhodamine-(5-(and-6))-isothiocyanate (TRITC) (Molecular Probes, Eugene, Oregon, USA) according to the manufacturer's instructions. TRITC-labeled spores were filtered through Steriflip Filter Units (Millipore, Cork, Ireland), aliquoted, and stored at 4°C until use. Viability tests were performed by inoculating an aliquot of stained conidia onto an AMM agar plate and incubating for 3 days at 37°C.

### Induction of AAI

BALB/c mice were sensitized with 10 μg OVA Grade VI (Sigma-Aldrich) adsorbed to 1.5 mg of Imject alum (Thermo Scientific, Rockford, IL, USA) diluted in PBS. OVA inoculation was carried out on days 0, 14, and 21 via intraperitoneal (i.p.) injection. The animals were then challenged with 1% OVA aerosol in PBS for 20 minutes on day 27 (OVA/OVA). The negative control groups (OVA/PBS) were exposed to PBS instead of OVA aerosol.

### Application of *A. fumigatus* conidia

At the acute stage of AAI, 12 hours after single OVA aerosol challenge, mice received 5 × 10^6^ TRITC-labeled *A. fumigatus* conidia/mouse (n = 3–5 per group per time point). Mice were anesthetized with 2-bromo-2-chloro-1,1,1-trifluoroethane (Sigma-Aldrich). Conidia were dissolved in PBS to a concentration of 1 × 10^8^ conidia/mL and administrated to the oropharyngeal cavity of mice [22] in a total volume of 50 μL. Then mice were subjected to either BAL collection or whole-mount airway dissection.

### BAL collection

Animals were lethally anesthetized with an overdose of i.p.-administered pentobarbital (Narcoren; Rhone Merieux, Laupheim, Germany) immediately before *A. fumigatus* conidia application (0 hours), and at 2, 4, 8, and 24 hours post-application of conidia. BAL was performed twice with 0.8 mL of ice-cold sterile PBS. Cells were quantified using a Goryaev-chamber (Minimed, Suponevo, Bryansk, Russia), and diluted to a concentration of 1 × 10^6^ cells/mL. Cells were then transferred onto glass slides using a Cytospin 2 centrifuge (Shandon, London, UK). Cells were stained using a Diff-Quik stain set (Diachem, Saint-Petersburg, Russia), and the differential cell counts were assessed. Conidia that were internalized by BAL phagocytic cells and non internalized conidia were counted.

### Tissue processing and whole-mount immunostaining

The tissue was processed as described earlier [23]. Briefly, animals were lethally anesthetized as described above; the lungs were inflation-fixed with 2% paraformaldehyde (Sigma-Aldrich), and the main axial pathways of each lobe were microdissected. The airways were then washed with PBS for 1 hour and permeabilized with 0.3% Triton X-100 in PBS for 2 hours. Washings with PBS (3 times for 10 minutes) accompanied each step of the staining. Bronchi were then blocked with 1% BSA in PBS and immunostained as whole-mounts. The left lung and the right inferior lobe were used for specific antibody staining analysis, and the right middle and post-caval lobes were used for isotype controls (Additional file 1: Figure S1). All antibodies were diluted in PBS supplemented with 0.5% BSA and 4% mouse, rat, or donkey serum according to the antibody type. Directly FITC-conjugated rat monoclonal anti-mouse Ly-6G antibody (clone RB6-8C5; eBioscience, San Diego, CA, USA) and primary rat anti-mouse I-A/I-E antibody (clone M5/114.15.2; Biolegend, San Diego, CA, USA) were used at a dilution of 1/50. The Cy5-conjugated donkey anti-rat IgG secondary antibody (Jackson Immuno Research, West Grove, PA, USA) was used at a dilution of 1/400. Finally, all samples were mounted in Prolong Gold mounting medium (Molecular Probes, Eugene, OR, USA).

### Confocal microscopy

Images were acquired using an LSM 510 META (Carl Zeiss, Jena, Germany) confocal microscope using 20× and 40× (water immersion) objectives. Laser wavelengths of 488 nm, 543 nm, and 633 nm were used for the excitation of the fluorochromes FITC, TRITC, and Cy-5, respectively. Specimens were scanned in Z-stack "channel/multi-tracking" mode with the appropriate emission filters. For quantitative analysis, the image stacks containing the airway epithelium and smooth muscle layer were scanned with an XYZ-resolution of 1024 × 1024 × 70, with dimensions of 318.2 μm × 318.2 μm × 39.81 μm, respectively. Airways were divided into four segments: two proximal (dorsal and ventral), and two distal (dorsal and ventral). At least two image stacks were taken at each segment, one on the ventral and one on the dorsal side of the main axial pathway. Preference was given to locations containing a minimum of one *A. fumigatus* conidium. Confocal images were shown as two-dimensional maximum intensity projections (MIPs) or three-dimensional surface objects. The final image processing was carried out using Adobe Photoshop CS version 5 software (Adobe Systems, Mountain View, CA, USA).

### Quantitative image analysis

Image stacks were analyzed using Imaris 6.2.1 software developed by Bitplane (Zurich, Switzerland). Surface rendering was performed using optimal threshold settings in the Ly-6G (neutrophils), *A. fumigatus* conidia, and MHC II (APC) channels by "region growing" [23]. Threshold and filter settings were optimized by visually comparing the result with the MIP. Cell numbers were automatically calculated from the respective surface objects. To quantify the number of conidia taken up by neutrophils and APCs, in the *A. fumigatus* conidia channel the mean intensity filter was adjusted for the Ly-6G^+^ and MHC II^+^ channels, respectively. To identify Ly-6G^+^ and MHC II^+^ cells that had internalized conidia, cells with a mean fluorescence intensity above a certain threshold in the *A. fumigatus* conidia channels were selected. Visual control of selection regions was carried out.

### Statistical analysis

Data are expressed as mean ± SEM. To compare groups of mice, and airway segments 2-way ANOVA test and Bonferroni post-test were applied. Cell and conidia numbers or ratios between the indicated time point and the initial time point were compared using one-way ANOVA and Dunnett's multiple comparison tests. Tests were performed using GraphPad Prism version 4.03 for Windows (GraphPad Software, San Diego, CA, USA). Differences with p < 0.05 were considered statistically significant.

## Results

### Contribution of different BAL phagocytic cells to *A. fumigatus* conidial internalization in mice with AAI

**Figure 1:**
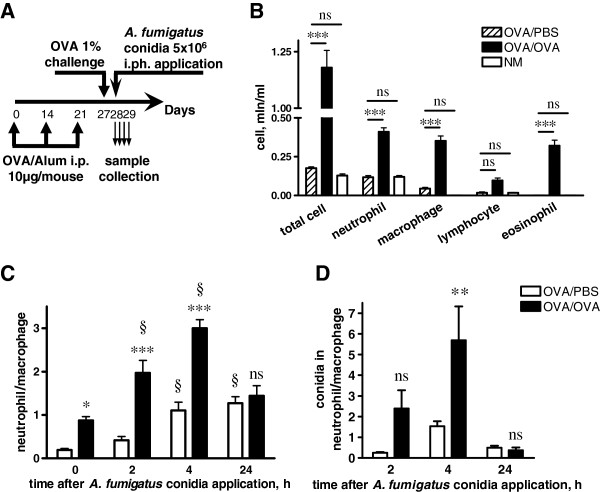
**Internalization of *A. fumigatus *conidia by BAL cells during AAI. (A)** Mice received three i.p. injections of OVA (on days 0, 7, and 21; 10 μg/mouse/injection), and were challenged on day 27 with OVA (1% in PBS) (OVA/OVA group) or PBS (OVA/PBS group). Mice were inoculated with *A. fumigatus* conidia via inhalation at 24 hours post-allergen challenge. BAL samples were analyzed at 0, 2, 4, and 24 hours post-conidial administration. **(B)** Total and differential cell numbers immediately before conidia application were evaluated. NM – non-treated mice. **(C)** The neutrophil to macrophage ratio in BALs of OVA/PBS (open bars) or OVA/OVA (black bars) mice at the indicated time points following conidial application. **(D)** The ratio of internalized by neutrophils to internalized by macrophages conidia numbers at the different time points following conidial application to OVA/PBS (open bars) and OVA/OVA (black bars) mice. Data are shown as means ± SEM for two representative experiments, with three and five mice per group. OVA/OVA versus OVA/PBS group or OVA/PBS versus NM: * (p < 0.05), *** (p < 0.001), and ns: not significant. Neutrophil to macrophage ratio at the indicated time point versus the time point immediately before conidial application: § (p < 0.01).

In the present study, we used a well-established OVA-induced mouse model of AAI. Mice were inoculated with *A. fumigatus* conidia via inhalation at the acute phase of AAI, 12 hours post-single allergen challenge (Figure 1A). To mimic the resting state of conidia, and to exclude an activation of the immune system as a result of conidial germination, freshly harvested conidia were fixed with paraformaldehyde. The control inoculation revealed the absence of conidial growth within 72 hours of incubation at 37°C.

The total leukocyte recruitments and differential BAL cell compositions were compared for OVA/PBS, OVA/OVA, and intact mice 12 hours following the allergen inhalation immediately before conidia application. Both total cell number and the number of macrophages, neutrophils, eosinophils, and lymphocytes elevated significantly in the BAL of mice with acute phase AAI (Figure 1B).

The numbers of macrophages, neutrophils, eosinophils, lymphocytes, and *A. fumigatus* conidia in the BALs of mice were quantified at 2, 4, and 24 hours post-application of conidia. We used the ratios of neutrophils to macrophages to identify the prevalence of the respective phagocytes at different time points. Instilled conidia induced neutrophil recruitment in both OVA/PBS and OVA/OVA mice (Figure 1C, Additional file 2: Figure S2A,B), but only in OVA/OVA group neutrophils outnumbered macrophages (Figure 1C). The percentage of conidia internalized by BAL phagocytes referred to hereafter as the ingestion potential of the phagocytes, and the ratios of neutrophil ingestion potentials to these of macrophages indicated the dominance of neutrophils or macrophages in conidia internalization at the different time points. We observed the correlation between the number and ingestion potential of BAL neutrophils in mice with AAI. Thus, at 4 hours after conidial application, the ingestion potential of neutrophils significantly exceeded that of alveolar macrophages in OVA/OVA animals (Figure 1D, Additional file 2: Figure S2D) but not in OVA/PBS mice (Figure 1D, Additional file 2: Figure S2C). At 24 hours post-inhalation of conidia, both the percentage (Figure 1C) and the ingestion potential (Figure 1D) of BAL neutrophils decreased, and macrophages became the cells predominantly responsible for conidial internalization in both OVA/PBS and OVA/OVA mice (Figure 1C,D, Additional file 2: Figure S2C,D). These results demonstrated that within 24 hours, BAL phagocytic cells internalized more than 80% of aspirated *A. fumigatus* conidia. Consequently, the majority of conidia was captured, and therefore could not penetrate the epithelial barrier and disseminate into the airway tissues.

### Visualization of *A. fumigatus* conidia, neutrophils, and MHC II^+^ APCs in the conducting airway mucosa

**Figure 2:**
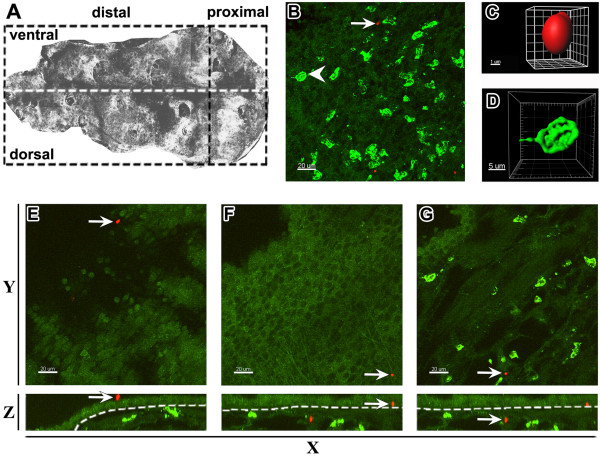
**Distribution of *A. fumigatus *conidia and Ly-6G**^**+ **^**neutrophils along the mouse conducting airway.** The conducting airway was dissected from the right inferior lung lobe of intact mice that received 5 × 10^6^*A. fumigatus* conidia 4 hours prior to dissection. **(A)** Microdissected mouse airway represented as montage of the number of confocal images scanned with low magnification from the luminal side of the specimen was arbitrarily separated (dashed lines) into proximal, distal, dorsal, and ventral regions. **(B)** Representative three-dimensional image showing the distribution of Ly-6G^+^ neutrophils (green) and TRITC-labeled *A. fumigatus* conidia (red) in the conducting airway mucosa. **(C)** The single conidium and **(D)** neutrophil indicated in **(B)** are represented as the three-dimensional objects. **(E)** The conidium located on the luminal side of the conducting airway epithelium, **(F)** in the subepithelial area, and **(G)** parenchymal tissue, is indicated by arrows on Z-projections **(**top view: **E**, **F**, **G** upper panels**)** and Y-projections **(**front view: **E**, **F**, **G** lower panels**)**. Epithelial and subepithelial compartments were separated based on epithelium auto-fluorescence **(**dashed line on **E**, **F**, **G** lower panels**)**. Scale bar = 20 μm **(B**, **E**, **F**, **G)**; scale bar and greed spacing = 1 μm **(C)**, and 5 μm **(D)**.

Upon inhalation, *A. fumigatus* conidia were disseminated throughout the conducting airways and penetrated the small airways [17]. The airway mucosa is the primary site of interactions between conidia and host immune cells; therefore, we focused on precise analysis of the conducting airway mucosa. To detect the location of conidia along the length of the intrapulmonary (bronchial) conducting airway, we administrated TRITC-labeled conidia to the pharyngeal cavity of mice. Confocal images of whole-mounted conducting airways enabled us to identify the distribution of conidia at the proximal and distal segments of the conducting airways (Figure 2A). High-resolution confocal image stacks served as a basis for the generation of three-dimensional surface objects, which were used to detect single conidia both in the epithelial compartment and subepithelial area of the airway mucosa. We identified conidia as globular objects with an approximate diameter of 3 μm (Figure 2B,C). Quantitative analysis of the surface object distribution showed no preferential localization of conidia at any specific anatomical location; TRITC-labeled spores were scattered along the airway and distributed randomly within both proximal and distal airway segments (Additional file 3: Figure S3).

Neutrophils were identified in conducting airways using anti Ly-6G staining. Ly-6G (also known as Gr-1 antigen) is a GPI-linked myeloid differentiation marker that is located on the outer side of the cell plasma membrane. Gr-1 is also expressed on plasmacytoid DCs, but this population is poorly represented in the mouse conducting airways [24,25]. The three-dimensional surface rendering of the high resolution confocal images revealed a specific surface contour (spheroid object with several cavities) of Ly-6G positive cells (Figure 2B,D).

Conidia administrated to intact mice were detected not only on the luminal side of the epithelium (Figure 2E, red), but also beneath the epithelium (Figure 2F, red), and even in the lung tissue (Figure 2G, red). Neutrophils from control mice generally did not penetrate the epithelial barrier, and occupied the subepithelial compartment and parenchymal tissue (Figure 2E-G, green).

To identify conducting airway APCs, which can also take part in internalization of conidia, we used anti-MHC II staining. Using the findings of Veres et al. [26], we identified two populations of conducting airway mucosal MHC II^+^ APCs, which were distinguished by the morphology and anatomical location: an epithelial (or intraepithelial) DC population, and a population containing subepithelial DCs (Additional file 4: Figure S4A,D,G and C,F,I: epithelial DCs, arrows; subepithelial DCs, arrowheads). Although several studies using flow cytometry of peripheral blood and lymph node leukocyte samples have reported that neutrophils are capable of expressing MHC class II [27], we did not identify expression of MHC II molecules on the surface of Ly-6G^+^-conducting airway neutrophils by confocal microscopy of the main bronchus specimen (Additional file 4: Figure S4B,E,H,C,F,I: MHC II, yellow; Ly6-G, green).

### Distribution of neutrophils recruited to conducting airway mucosa of mice with AAI in response to *A. fumigatus* conidia application

To investigate the difference in antifungal neutrophil activity between AAI and control mice at 24 hours post-challenge, both groups were inoculated with *A. fumigatus* conidia via inhalation. We compared the spatial location of neutrophils in the conducting airways of OVA/OVA and OVA/PBS mice shortly after conidial application.

**Figure 3:**
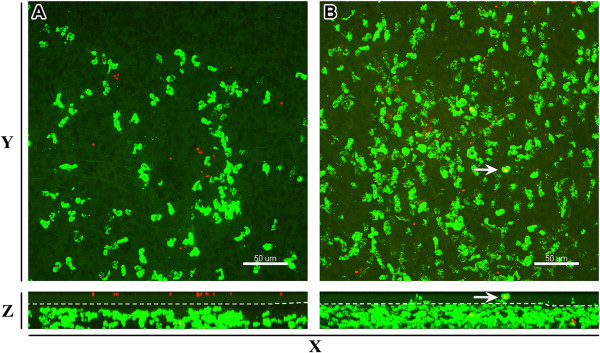
***A. fumigatus *conidia-induced recruitment of neutrophils to the conducting airways of mice with AAI.** Representative confocal images taken from the distal ventral segment of conducting airway at 2 hours post-conidial inhalation by **(A)** OVA/PBS and **(B)** OVA/OVA mice. Images are represented as Z- (upper panels) and Y-projection (lower panels). Ly-6G^+^ neutrophils are shown in green and epithelial auto-fluorescence in dark green. Epithelial and subepithelial compartments, separated by a dashed line, were determined based on epithelial auto-fluorescence (lower panels). Neutrophil in the epithelial compartment of the conducting airway mucosa is indicated by arrow. The images are shown as MIP and the scale bar = 50 μm.

The results demonstrated that in the absence of preexisting AAI, Ly-6G^+^ neutrophils located strongly in the subepithelial compartment of the conducting airway mucosa (Figure 3A). In contrast to the OVA/PBS group, neutrophils from OVA/OVA mice were detected in close proximity to the epithelium (Figure 3B), as well as in the epithelial compartment of the airway mucosa (Figure 3B; arrow).

### Internalization of *A. fumigatus* conidia by conducting airway phagocytic cells

**Figure 4:**
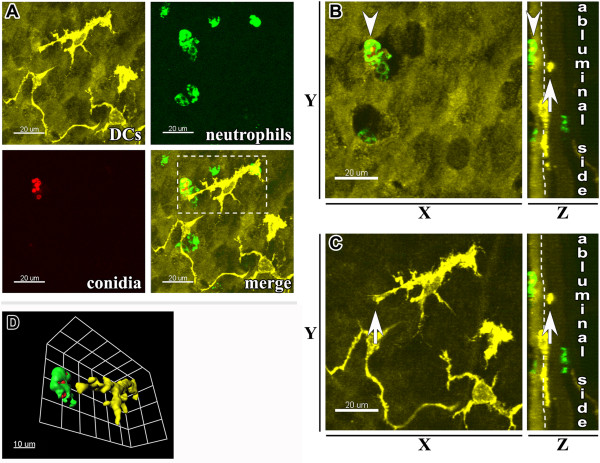
**Internalization of *A. fumigatus *conidia in the epithelial compartment of conducting airways. (A)** Representative confocal image taken from the proximal ventral segment of the whole-mount airway main axial pathway showing the distribution of MHC II^+^ epithelial DCs (upper left panel: MHC II^+^ DCs, yellow; epithelium auto-fluorescence, dark yellow), Ly-6G^+^ neutrophils (upper right panel, green), *A. fumigatus* conidia (lower left panel, red), and a merged image (lower right panel) at 4 hours post-conidial application. Scale bar = 20 μm. **(B**, **C)** The neutrophil locating in close proximity to the luminal side of the epithelium (arrowhead) and the intraepithelial DC (arrow) are indicated on Z-projection (left panels) or on X-projection (right panels) of the area displayed in **(A)**. **(D)** The same neutrophil and DC are displayed on the three-dimensional larger magnification image of the region boxed in **(A)**. Scale bar = 20 μm **(A**, **B**, **C)**; and 10 μm **(D)**.

We then analyzed the contribution of Ly-6G^+^ neutrophils and MHC II^+^ APCs to internalization of *A. fumigatus* conidia. In response to the allergen and conidial inhalation, neutrophils trafficked to the subepithelial and intraepithelial areas of conducting airways, and migrated through the epithelium. These neutrophils were predominantly observed in close proximity to the luminal side of the airway epithelium (Figure 4A: upper right; B: arrowhead). Neutrophils became activated following transepithelial migration and internalized conidia, thereby preventing contacts between the epithelial DCs and conidia (Figure 4A-D). Epithelial DC extensions were commonly observed to be projected toward *A. fumigatus* conidia; however, precise analyses of epithelial DC-neutrophil-conidia interactions revealed the absence of direct contact between epithelial DCs and neutrophils or conidia (Figure 4D).

**Figure 5:**
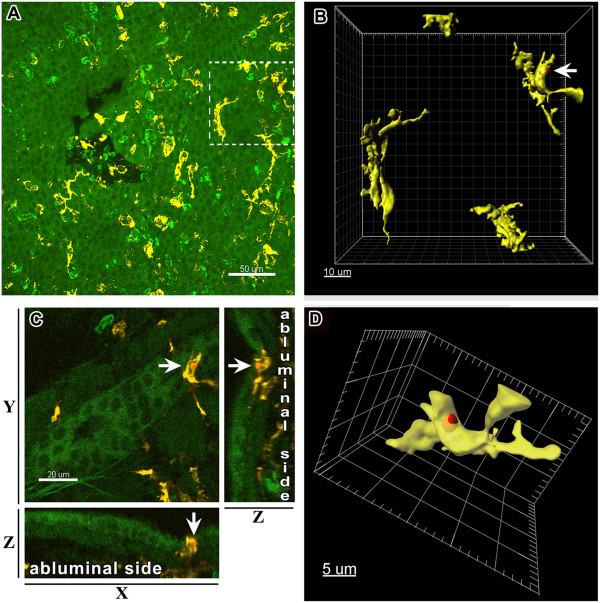
**Capture of conidia by the conducting airway epithelial DCs. (A)** Three-dimensional reconstitution of the image stacks taken from the proximal ventral region of the conducting airway showing the distribution of Ly-6G^+^ neutrophils (bright green), epithelium auto-fluorescence (dark green), MHC II^+^ APCs (yellow), and *A. fumigatus* conidia (red) in an OVA/PBS mouse at 8 hours post-conidial application. Scale bar = 50 μm. **(B)** Three-dimensional reconstitution of the area indicated on **(A)** representing MHC II^+^ APCs (yellow) and *A. fumigatus* conidia (red: arrow); scale bar = 10 μm. **(C)** X, Y, and Z-projections of the area framed in **(A)** showing the precise location of an intraepithelial (epithelium auto-fluorescence, green) and epithelial DC (yellow) that internalized *A. fumigatus* conidia (red: arrow). Scale bar = 20 μm. **(D)** An epithelial DC that internalized conidia is represented as the enlarged and arbitrary rotated three-dimensional surface object. Scale bar = 5 μm.

We observed the internalization of conidia by epithelial DCs is just three out of more than 200 analyzed samples (Figure 5A-D). All examples of immediate interaction between MHC II^+^ cells possessing long cellular extensions and conidia were detected in OVA/PBS mice at 8 hours post-application of conidia. These interactions we not observed in OVA/OVA animals.

We visualized free conidia and conidia captured by phagocytic cells in the subepithelial area of the conducting airway. Both neutrophils and subepithelial DCs took part in conidial internalization (Additional file 5: Figure S5A,B).

### Quantitative analyses of conidial internalization by the conducting airway neutrophils and APCs

To estimate the contribution of the different types of conducting airway phagocytic cells to dormant *A. fumigatus* conidial internalization, the number of ingested conidia and the ingestion rate were quantified for Ly-6G^+^ neutrophils and MHC II^+^ APCs.

As the total number of conidia (internalized by the phagocytes and non-internalized) in the conducting airways of both OVA/OVA and OVA/PBS mice did not vary significantly within 24 hours post-application (Additional file 6: Figure S6A), the percentage of conidia internalized by neutrophils or APCs reflected their ingestion effectiveness.

**Figure 6:**
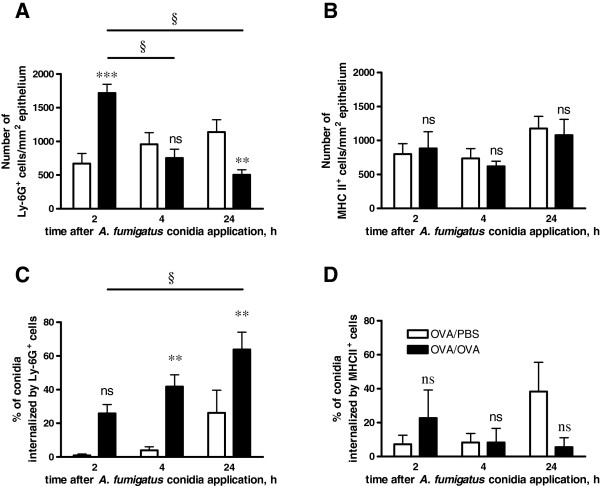
**Quantitative analysis of the number and ingestion effectiveness of conducting airway neutrophils and APCs.** The numbers of conducting airway Ly-6G^+^ neutrophils **(A)** and MHC II^+^ APCs per mm^2^ of epithelium **(B)** and the percentages of conidia that were internalized by neutrophils **(C)** and APCs from the total number of conidia in the region under observation **(D)** were detected. Data were acquired for OVA/OVA (black bars) or OVA/PBS (open bars) animals at different time points following *A. fumigatus* conidial application. Mean and SEM are presented for two independent experiments with three and four mice per group, respectively. The difference between OVA/OVA and OVA/PBS groups: ** (p< 0.01), *** (p< 0.001), and ns: not significant. Significant difference between neutrophil numbers and conidia percentages at initial and indicated time points: § (p<0.01).

The transient influx of neutrophils to the subepithelial area of conducting airways was observed in mice with AAI at 2 hours post-application of *A. fumigatus* conidia (Figure 6A). At 4 hours post-application the number of Ly-6G^+^ cells in the OVA/OVA group decreased, and reached a level similar to that in control mice (Figure 6A). At 24 hours post-application, the number of neutrophils in the conducting airways of OVA/OVA mice was significantly lower than that in the OVA/PBS group (Figure 6A). However, this significant reduction in neutrophil number did not reflect the ability of neutrophils to uptake the conidia (Figure 6C, Additional file 6: Figure S6B,D). Although the cell number subsequently decreased, the ingestion potential of neutrophils in OVA/OVA mice increased, and significantly exceeded that of the respective control group at all time points following conidial inhalation (Figure 6C, Additional file 6: Figure S6F).

Inhalation of conidia did not affect the total population of conducting airway DCs in either the OVA/OVA or OVA/PBS mice. There were no cardinal alterations in the total number (Figure 6B) and ingestion potential (Figure 6D, Additional file 6: Figure S6C,E,G) of conducting airway MHC II^+^ APCs at any of the analyzed time points.

## Discussion

This study investigated how exposure to respiratory allergens affects the ability of phagocytic cells to control the dissemination of routinely inhaled corpuscular antigens, particularly resting *A. fumigatus* conidia, in the airway. We observed that allergen inhalation induced transient neutrophil recruitment at the acute phase of AAI, and that conidia introduced during the acute phase induced recurrent neutrophil infiltration. Neutrophils were primarily responsible for the internalization of the dormant fungal spores in both BAL and conducting of mice with AAI. In the absence of pathogen inhalation or repetitive airborne allergen exposure, neutrophil recruitment to the airway, along with their ingestion activity, was terminated. Therefore, the ingestion potential of BAL macrophages significantly increased at 24 hours post-application of conidia. While we observed a correlation between the number and ingestion capacity of BAL neutrophils, conducting airway neutrophils of AAI mice showed an enhanced ability to internalize conidia even though the neutrophil number decreased.

Neutrophils are not considered to be a major factor in AAI. However, increased numbers of airway neutrophils have been observed in both human and animal studies of allergic asthma [18,19,28,29]. Consistent with these previous findings, we showed that a single exposure to an allergen (OVA) induces massive neutrophil infiltration to the airways shortly after allergen challenge. Such transient neutrophil influx is likely to be induced by allergen-specific antibodies or contamination of allergen with endotoxin [30-32].

Neutrophils are the most important cells in early antifungal defense [14,17]. In our model, the application of *A. fumigatus* conidia at the acute phase of AAI provoked *de novo* transient neutrophil recruitment, although the number of neutrophils in the airways was still increased. Because neutrophils migrate through the subepithelial area of conducting airways to reach the airway lumen, we observed simultaneous increases in neutrophil numbers in the BAL and conducting airways of mice in response to both allergen and conidial inhalation. However, we observed an earlier decrease in neutrophil numbers in conducting airways, which was followed later by a similar decrease in the BAL samples. Our observation confirms the one-way transepithelial traffic of neutrophils that was suggested previously [33]. Thus, in the absence of recurrent inhalation of exogenous irritants, the neutrophilic inflammation was resolved, as is the ability of BAL neutrophils to internalize dormant fungal spores. Notably, in BALs that were taken from mice 24 hours after conidial application, macrophages showed a greater contribution to conidial internalization than neutrophils, which highlights the major role of macrophages in the resolution of inflammation.

Because of their small size (3 μm), *A. fumigatus* conidia can penetrate the small airways and reach the alveoli [34,35]. Therefore, the primary cells encountering the conidia were assumed to be alveolar macrophages [36]. However, Krenke et al. [37] observed that the tracheobronchial manifestations of *Aspergillus* infections could suggest that immune cells can sense conidia before they reach the alveoli. In the present study, we used three-dimensional mapping to investigate conidial distribution in conducting airway mucosa. We found that instilled conidia penetrated the airway epithelial border, and were present both on the luminal side of the bronchial epithelium and in the subepithelial area of conducting airways shortly after the inhalation. As alveolar macrophages do not reside at the luminal side of the bronchial epithelium, other immune cells must be responsible for fungal spore recognition and neutralization in the conducting airway compartment. Because of the specific location of airway mucosal DCs, they have been assumed to sense exogenous antigens in the airway lumen [38,39]. *In vivo* studies using triple cell co-cultures or silicone matrixes have shown that monocyte-derived DCs can internalize corpuscular antigens of 1 μm or larger [40,41]. However, *ex vivo* results of both the present study and the investigations of Veres et al. [26] provide evidence that conducting airway epithelial DCs sense corpuscular antigens (*E. coli* particles, polystyrene beads, or *A. fumigatus* conidia) without immediate contact. Our data revealed that conducting airway epithelial DCs can occasionally take up conidia, but not in mice that exhibited AAI. This finding may result from an enhanced neutrophil ingestion activity that was observed in conducting airways of challenged OVA mice.

We demonstrated that allergen challenge activated the transepithelial migration, followed by the massive neutrophil recruitment to the airways. Subsequently, on the luminal side of the conducting airway epithelium, neutrophils captured conidia and thereby prevented epithelial DC-conidia contacts. Although epithelial DC-conidial interactions in conducting airways rarely occur, the phenomenon should be investigated further.

In contrast to the epithelial DCs, spherical MHC II^+^ APCs captured conidia in the subepithelial space. Nevertheless, these APCs had a lower contribution than neutrophils in conidial internalization in both AAI and control mice. Thus, our data indicated that along with alveolar macrophages, conducting airway neutrophils are the primary cells to encounter aspirated *A. fumigatus* conidia.

In addition, while more than 80% of *A. fumigatus* conidia were internalized by BAL phagocytes only about 50% of spores were internalized by the joint efforts of conducting airway neutrophils and APCs 24 hours after the application of conidia to both OVA/OVA and OVA/PBS mice. The fungal spores that remained unbound by the main phagocytes can hide in the airway tissue until favorable for germination condition and therefore they can be considered as a life-threating factor.

To summarize, we demonstrated that application of conidia at the acute phase of AAI induces a massive neutrophil influx to conducting airways, bringing the migrated cells into close proximity with the conducting airway epithelium, followed by transmigration to the luminal side. The more than two-fold increase in neutrophil number indicated an intensification of the process of neutrophil-conidial interaction and spore internalization. Surprisingly, when the number of neutrophils in the conducting airways of mice with AAI reduced, the effectiveness of ingestion by these neutrophils remained significantly higher than in control mice. In addition, the increased ability to internalize corpuscular antigens was caused by the activated state rather than by the number of conducting airway neutrophils. *In vitro* studies have shown that internalization of fungal spores is crucial for conidia killing [17]. On the other hand, internalization of resting conidia and subsequent degradation of the hydrophobic surface layer in phagolysosomes enables their recognition by the intracellular pattern recognition receptors and initiates inflammatory response [9,42]. Hence, the allergen-induced enhancement of the capacity of conduction airway neutrophils (that we observed in mice with AAI but not in control animals) may indicate a break in tolerance to resting fungal spores that is normally exhibited by immunocompetent hosts [8]. Epidemiologic studies have associated sensitivity to *Aspergillus* fungi with the severe persistent asthma in adults [2]. Thus, the allergen-induced skewing from tolerance to sensitization can partially explain the potential of inhaled fungal spores to exacerbate asthma symptoms.

## Conclusions

In conclusion, our data show that at the acute phase of AAI, neutrophils are sufficient to control resting fungal spore dissemination. We demonstrated that the ingestion potential of neutrophils strongly depends on both the time and the location of the cell-pathogen interaction. Our data suggest that allergen-induced enhancement of the ingestion potential of conduction airway neutrophils may be a reason for the susceptibility of asthmatic patients to *Aspergillus* infection.

## Abbreviations

AAI: Allergic airway inflammation; AMM: *Aspergillus* minimal medium; APC: Antigen presenting cell; BAL: Bronchoalveolar lavage; Cy5: Cyanine 5; DC: Dendritic cell; FITC: Fluorescein isothiocyanate; GPI: Glycosylphosphatidylinisotol; i.p: Intraperitoneal; MHC: Major histocompatibility complex; MIP: Maximum intensity projection; OVA: Chicken ovalbumin; OVA/OVA mice: A group of mice that received intraperitoneal injection of ovalbumin and subsequent aerosol challenges with ovalbumin; OVA/PBS mice: A group of mice that received intraperitoneal injection of ovalbumin and subsequent aerosol challenge with PBS; PBS: Phosphate buffered saline; TRITC: Tetramethylrhodamine-(5-(and-6))-isothiocyanate.

## Competing interests

The authors declare that they have no competing interests.

## Authors’ contributions

MAS designed the study, performed the experiments, analyzed the data, and drafted the manuscript. ELB and EAS performed the experiments and analyzed the data. AMS drafted the manuscript. All authors read and approved the final manuscript.

## Supplementary Material

Additional file 1: Figure S1 Whole-mount immunostaining isotype control. Whole-mount conducting airways were immunostained with FITC-conjugated (A) monoclonal rat anti-mouse Ly-6G antibody to identify neutrophils or (B) rat IgG2b Isotype Control. To visualize MHC II^+^ APCs the other specimens were immunostained with primary (C) rat anti-mouse I-A/I-E antibody or (D) purified rat IgG2b followed by Cy5-conjugated donkey anti-rat IgG. Representative three-dimensional images showing epithelium auto-fluorescence (A, B, dark green; C, D, dark yellow), neutrophils (A, green), and APCs (C, yellow) were obtained as Z-stacks (30 optical slices × 50 μm) scanned from the luminal side of the airway. Scale bar = 20 μm. Click here for file

Additional file 2: Figure S2 Contribution of BAL macrophages and neutrophils to Internalization of *A. fumigatus* conidia. (A) Percentages of macrophages (open bars), and neutrophils (black bars) in BALs at the indicated time points following conidial application to OVA/PBS and (B) OVA/OVA mice. (C) Percentages of conidia that were internalized by macrophages (open bars), and neutrophils (black bars) at 2, 4, and 24 hours following conidial application to OVA/PBS and (D) OVA/OVA mice. Data are shown as means ± SEM for two representative experiments, with three and five mice per group. Significant difference between neutrophil and conidia percentages at the time point 0 and indicated time point: § (p<0.01). Click here for file

Additional file 3: Figure S3 Quantitative assay of *A. fumigatus* conidial distribution in the proximal and distal regions of conducting airways. The numbers of conidia were quantified for the proximal (open bars) and distal (black bars) regions of conducting airways of OVA/PBS mice at 2, 4, and 24 hours post-conidial-application. Mean and SEM are shown for two representative experiments with three and four mice per group. Statistical analyses revealed non-significant differences in conidia number between the airway segments at all analyzed time points. Click here for file

Additional file 4: Figure S4 Visualization of conducting airway MHC II^+^ APCs. Representative three-dimensional images of the proximal dorsal segment of the whole-mount conducting airway of an OVA/OVA mouse at acute stage of AAI. (A, D, G) MHC II^+^ APCs (yellow), (B, E, H) Ly-6G^+^ neutrophils (green) were visualized. (C), (F), and (I) Merged representations of the images shown in (A, B), (D, E), and (G, H), respectively. Confocal Z-stacks are represented as optical sections showing DCs and neutrophils in the (A, B, C) epithelial layer, (D, E, F) in close proximity to the epithelium, and (C, H, I) in the subepithelial area. (A, D, G, C, F, I) Structural cell auto-fluorescence is shown in dark yellow and (B, E, H) dark green. Epithelial and subepithelial DCs are indicated by arrows and arrowheads, respectively. Scale bar = 50 μm. Click here for file

Additional file 5: Figure S5 Internalization of *A. fumigatus* conidia in the subepithelial area of conducting airways by subepithelial DCs. Representative images of the proximal ventral segment of a whole-mount conducting airway excised from an OVA/PBS mouse at 4 hours post-conidial-application. The pictures are represented as X- (left panels) and Z-projections (right panels) showing interaction of (A) DCs (yellow) and conidia (red), or (B) neutrophil (green) and conidia (red). Conidium in contact to subepithelial DC is indicated by arrowhead; conidia inside neutrophil are indicated by arrow. Epithelial and subepithelial compartments were separated based on epithelium auto-fluorescence (dashed line). Scale bar = 20 μm. Click here for file

Additional file 6: Figure S6 Quantitative analysis of the number of *A. fumigatus* conidia, ingestion rate and ingestion capacity of neutrophils and APCs in conducting airways. The numbers of *A. fumigatus* conidia per 1 mm^2^ of conducting airway epithelium (A); the number of conducting airway Ly-6G^+^ neutrophils (B) and MHC II^+^ APCs (C) that internalized *A. fumigatus* conidia as well as the number of conidia that were internalized by Ly-6G^+^ neutrophils (F) and MHC II^+^ APCs (G) were quantified. The percentage of Ly-6G^+^ neutrophils (D) and MHC II^+^ APCs (E) that internalized conidia from the total number of the neutrophils and APCs respectively were calculated. Data were acquired for OVA/OVA (black bars) or OVA/PBS (open bars) animals at different time points following *A. fumigatus* conidial application. Mean and SEM are presented for two independent experiments with three and four mice per group, respectively. Significant difference between OVA/OVA and OVA/PBS groups: * (p<0.05) and ** (p< 0.01), *** (p< 0.001), and ns: not significant. Click here for file
