# Supplementary figures and images for: Elimination of Aspergillus fumigatus conidia from the airways of mice with allergic airway inflammation
Source: Respir Res. 2013 Jul 27;14(1):78. doi: 10.1186/1465-9921-14-78 (PMC3735401; doi:10.1186/1465-9921-14-78)

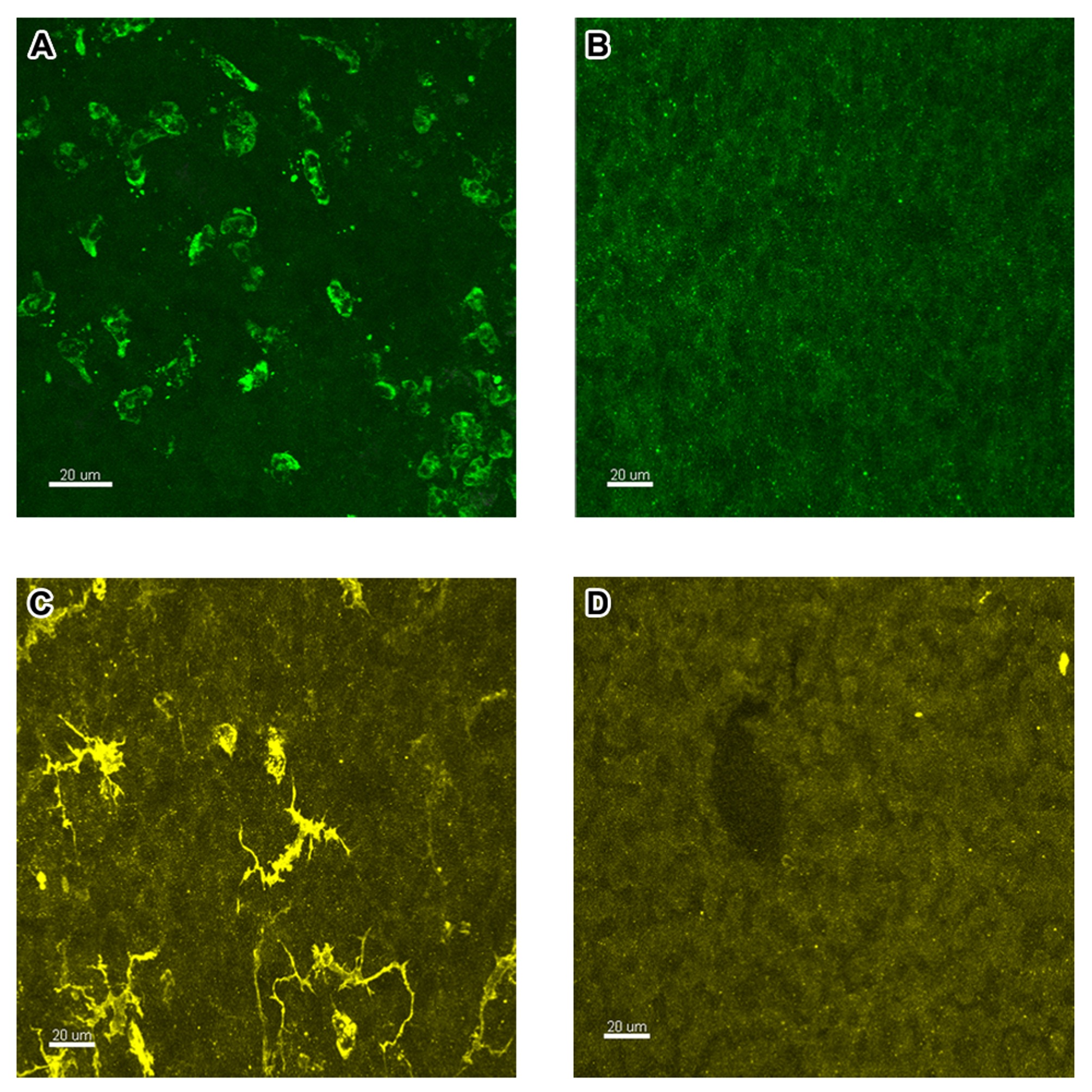

Supplement: Additional file 1: Figure S1 — Whole-mount immunostaining isotype control. Whole-mount conducting airways were immunostained with FITC-conjugated (A) monoclonal rat anti-mouse Ly-6G antibody to identify neutrophils or (B) rat IgG2b Isotype Control. To visualize MHC II+ APCs the other specimens were immunostained with primary (C) rat anti-mouse I-A/I-E antibody or (D) purified rat IgG2b followed by Cy5-conjugated donkey anti-rat IgG. Representative three-dimensional images showing epithelium auto-fluorescence (A, B, dark green; C, D, dark yellow), neutrophils (A, green), and APCs (C, yellow) were obtained as Z-stacks (30 optical slices × 50 μm) scanned from the luminal side of the airway. Scale bar = 20 μm. [file 1465-9921-14-78-S1.tiff]

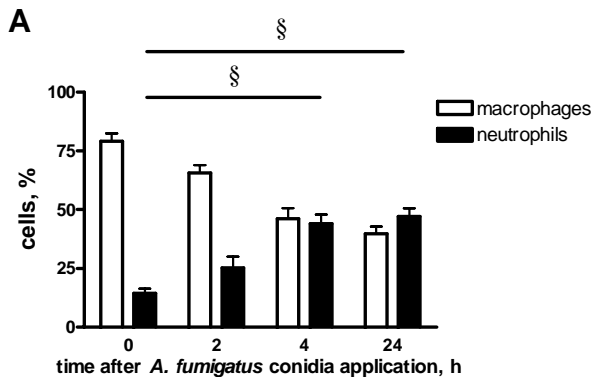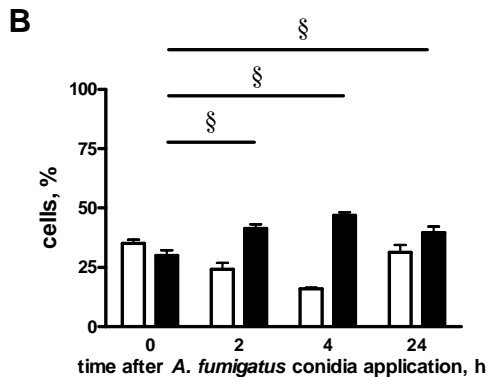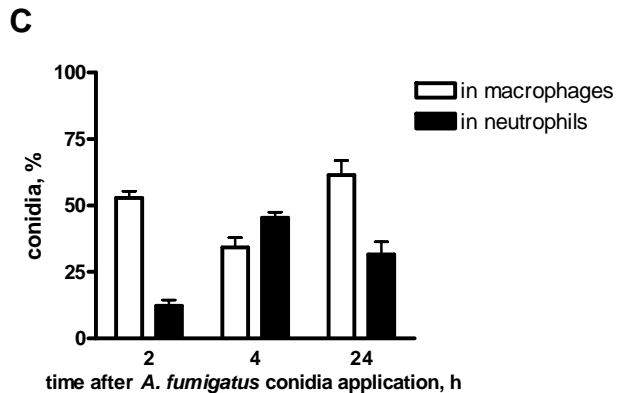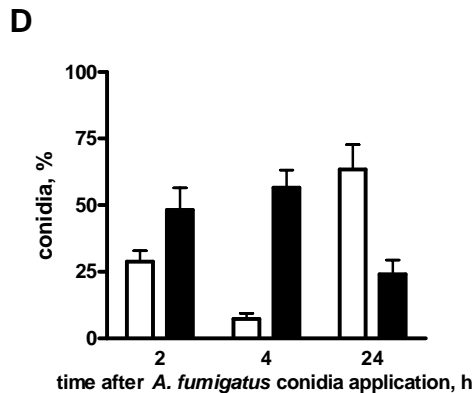

Supplement: Additional file 2: Figure S2 — Contribution of BAL macrophages and neutrophils to Internalization of A. fumigatus conidia. (A) Percentages of macrophages (open bars), and neutrophils (black bars) in BALs at the indicated time points following conidial application to OVA/PBS and (B) OVA/OVA mice. (C) Percentages of conidia that were internalized by macrophages (open bars), and neutrophils (black bars) at 2, 4, and 24 hours following conidial application to OVA/PBS and (D) OVA/OVA mice. Data are shown as means ± SEM for two representative experiments, with three and five mice per group. Significant difference between neutrophil and conidia percentages at the time point 0 and indicated time point: § (p<0.01). [file 1465-9921-14-78-S2.pdf]

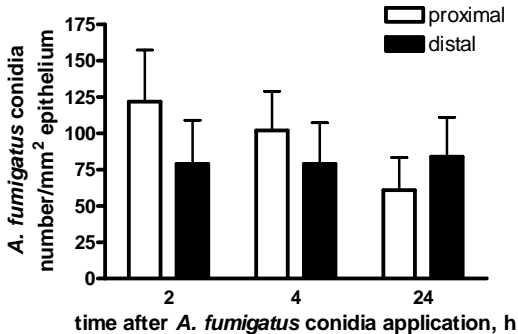

Supplement: Additional file 3: Figure S3 — Quantitative assay of A. fumigatus conidial distribution in the proximal and distal regions of conducting airways. The numbers of conidia were quantified for the proximal (open bars) and distal (black bars) regions of conducting airways of OVA/PBS mice at 2, 4, and 24 hours post-conidial-application. Mean and SEM are shown for two representative experiments with three and four mice per group. Statistical analyses revealed non-significant differences in conidia number between the airway segments at all analyzed time points. [file 1465-9921-14-78-S3.pdf]

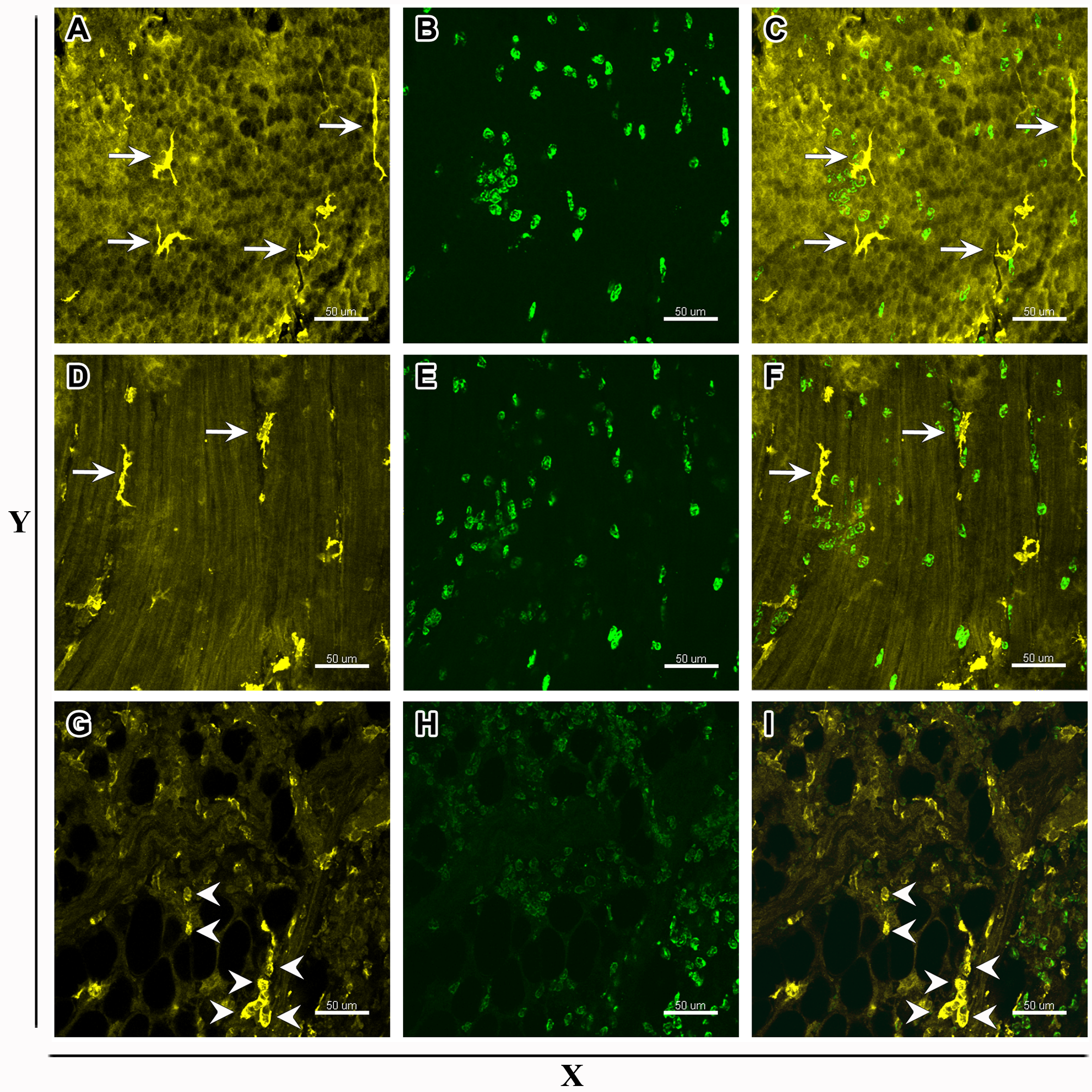

Supplement: Additional file 4: Figure S4 — Visualization of conducting airway MHC II+ APCs. Representative three-dimensional images of the proximal dorsal segment of the whole-mount conducting airway of an OVA/OVA mouse at acute stage of AAI. (A, D, G) MHC II+ APCs (yellow), (B, E, H) Ly-6G+ neutrophils (green) were visualized. (C), (F), and (I) Merged representations of the images shown in (A, B), (D, E), and (G, H), respectively. Confocal Z-stacks are represented as optical sections showing DCs and neutrophils in the (A, B, C) epithelial layer, (D, E, F) in close proximity to the epithelium, and (C, H, I) in the subepithelial area. (A, D, G, C, F, I) Structural cell auto-fluorescence is shown in dark yellow and (B, E, H) dark green. Epithelial and subepithelial DCs are indicated by arrows and arrowheads, respectively. Scale bar = 50 μm. [file 1465-9921-14-78-S4.tiff]

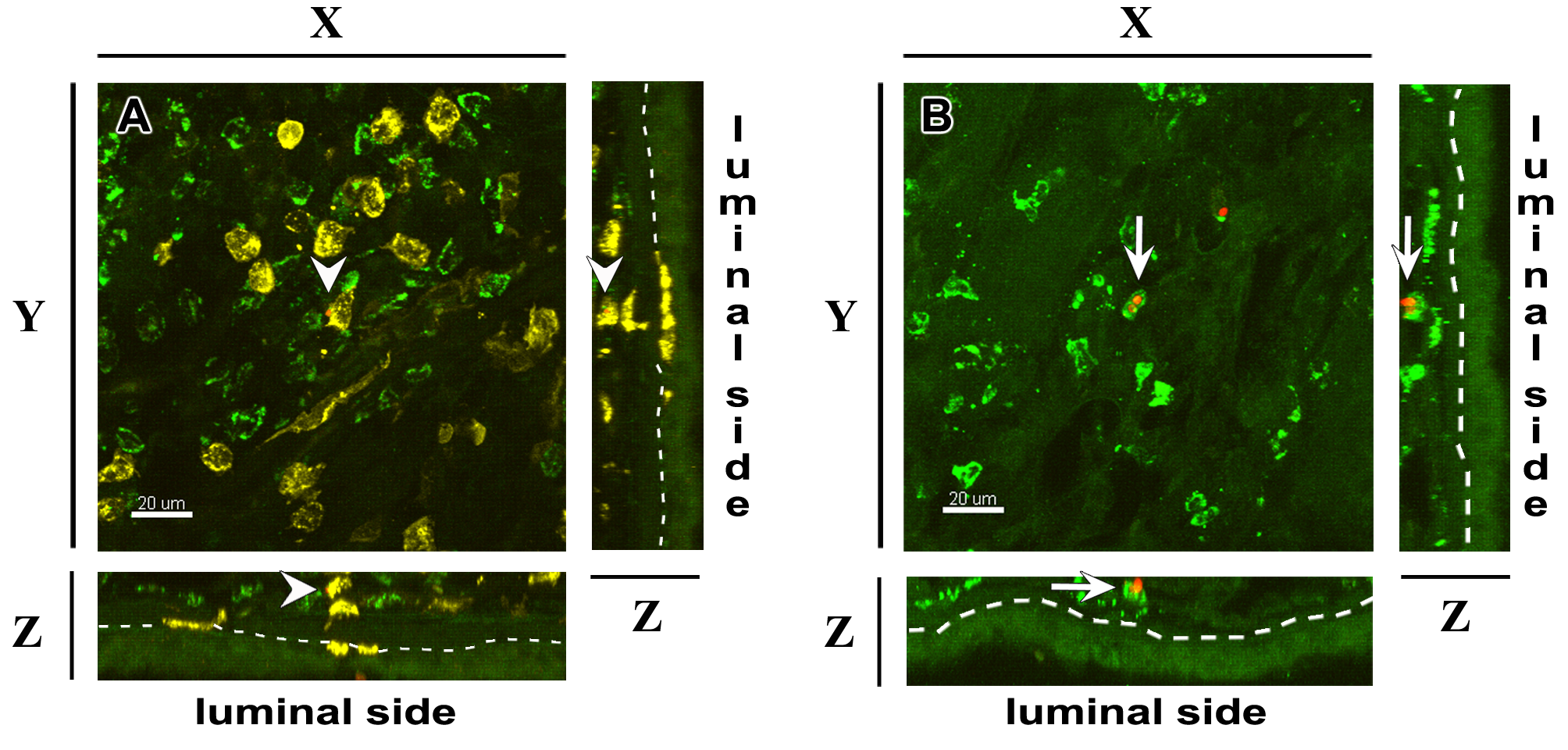

Supplement: Additional file 5: Figure S5 — Internalization of A. fumigatus conidia in the subepithelial area of conducting airways by subepithelial DCs. Representative images of the proximal ventral segment of a whole-mount conducting airway excised from an OVA/PBS mouse at 4 hours post-conidial-application. The pictures are represented as X- (left panels) and Z-projections (right panels) showing interaction of (A) DCs (yellow) and conidia (red), or (B) neutrophil (green) and conidia (red). Conidium in contact to subepithelial DC is indicated by arrowhead; conidia inside neutrophil are indicated by arrow. Epithelial and subepithelial compartments were separated based on epithelium auto-fluorescence (dashed line). Scale bar = 20 μm. [file 1465-9921-14-78-S5.tiff]

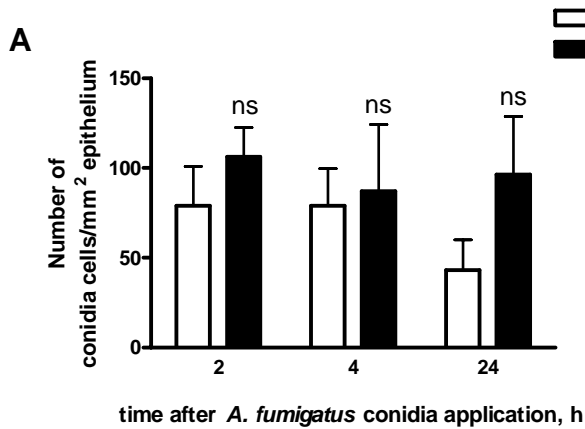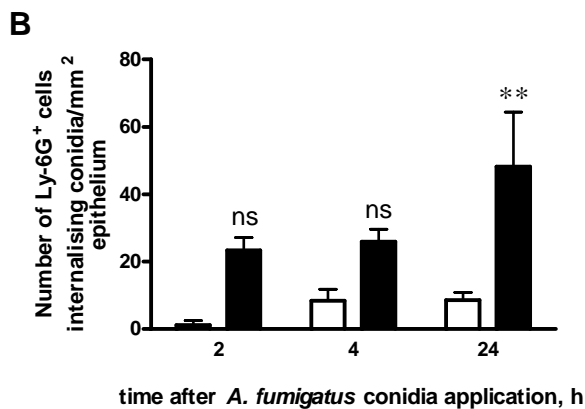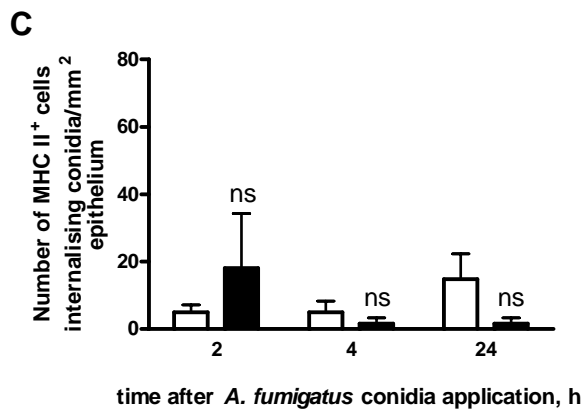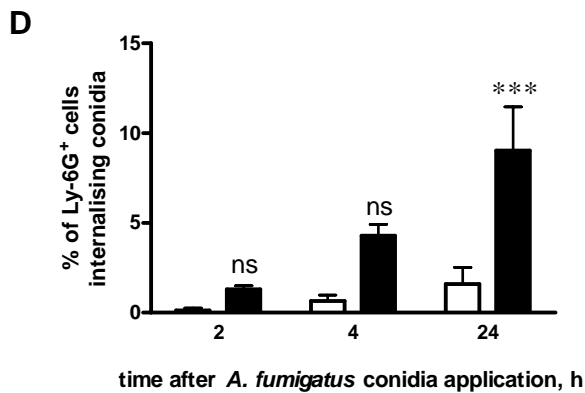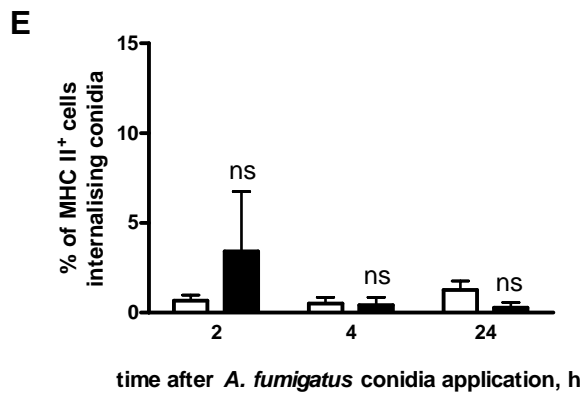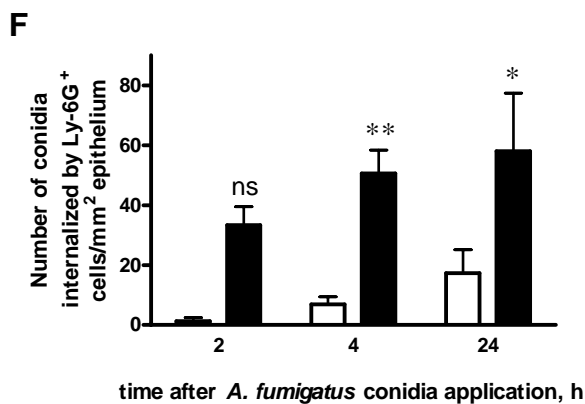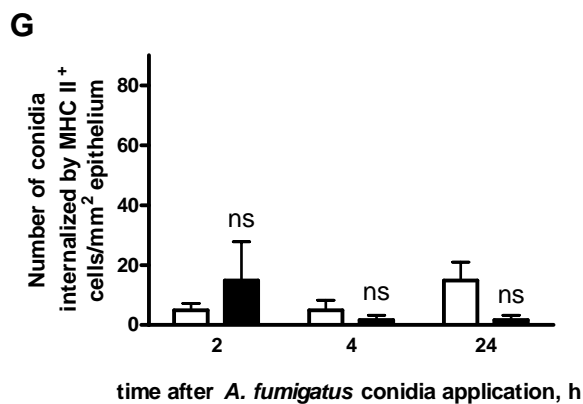

Supplement: Additional file 6: Figure S6 — Quantitative analysis of the number of A. fumigatus conidia, ingestion rate and ingestion capacity of neutrophils and APCs in conducting airways. The numbers of A. fumigatus conidia per 1 mm2 of conducting airway epithelium (A); the number of conducting airway Ly-6G+ neutrophils (B) and MHC II+ APCs (C) that internalized A. fumigatus conidia as well as the number of conidia that were internalized by Ly-6G+ neutrophils (F) and MHC II+ APCs (G) were quantified. The percentage of Ly-6G+ neutrophils (D) and MHC II+ APCs (E) that internalized conidia from the total number of the neutrophils and APCs respectively were calculated. Data were acquired for OVA/OVA (black bars) or OVA/PBS (open bars) animals at different time points following A. fumigatus conidial application. Mean and SEM are presented for two independent experiments with three and four mice per group, respectively. Significant difference between OVA/OVA and OVA/PBS groups: * (p<0.05) and ** (p< 0.01), *** (p< 0.001), and ns: not significant. [file 1465-9921-14-78-S6.pdf]
